# Nutritional composition, quality, and shelf stability of processed *Ruspolia nitidula* (edible grasshoppers)

**DOI:** 10.1002/fsn3.369

**Published:** 2016-04-13

**Authors:** Geoffrey Ssepuuya, Ivan Muzira Mukisa, Dorothy Nakimbugwe

**Affiliations:** ^1^ Department of Food Technology and NutritionSchool of Food TechnologyNutrition and Bio‐EngineeringCollege of Agricultural and Environmental SciencesMakerere UniversityP.O. Box, 7062KampalaUganda

**Keywords:** Acceptability, edible insects, nutritional value, processing, *Ruspolia nitidula*, shelf stability

## Abstract

The nutritional and commercial potential of the edible grasshopper (*Ruspolia nitidula*,* nsenene* in *Luganda*), a delicacy in Uganda and many East African tribes, is limited by a short shelf life and unverified nutritional value. This research established that *R. nitidula* is nutritious with 36–40% protein, 41–43% fat, 2.5–3.2% carbohydrate, 2.6–3.9% ash, 11.0–14.5% dietary fiber, and 900–2300 *μ*g/100 g total carotenoids on a dry matter basis. Sautéing was the most preferred processing method resulting in grasshoppers with a notably better aroma and flavor. After 12 weeks of storage at room temperature, processed and vacuum packed, ready‐to‐eat grasshoppers maintained their edible quality with an acid value of 3.2 mg KOH/g, a total plate count of log 1.8 cfu/g, and an overall acceptability of 6.7–7.2 on a 9‐point hedonic scale. Further research is required for extending the shelf stability beyond 12 weeks and characterizing the profile of major nutrients.

## Introduction

Entomophagy (the practice of eating insects) as well as their use in livestock and pet feeds are increasing worldwide (Durst et al. 2010; Van Huis et al. 2013; Bosch et al. 2014; Kenis et al. 2014; Kelemu et al. 2015). While edible insects were formerly consumed as a cultural delicacy mostly in developing countries, they are gaining recognition as important sources of nutrients (Belluco et al. 2013; Mlcek et al. 2014; Shockley and Dossey 2014).

Edible insects are highly nutritious. According to Capinera (2008) and a review of the nutritional composition of 236 edible insects (Rumpold and Schluter 2013), insects are high in energy, with 2–60% fat on a dry matter basis, which has a high proportion of mono‐ and polyunsaturated fatty acids, provide satisfactory protein (20–80%) which meets the human amino acid requirements, are high in minerals such as calcium, copper, iron, phosphorus, magnesium, manganese, and potassium, have an abundance of vitamin A and carotenoids, and though in low amounts, they can contain B vitamins such as riboflavin, pantothenic acid, and some times, folic acid. Using two nutrient profiling models developed to combat over‐ and undernutrition, Payne et al. (2015) concluded that insects’ contribution to health is not significantly lower than that of meat products, and can actually be significantly higher. Besides their potential contribution to dietary nutrient intakes, thus improving health, insects are also important for improving and conserving the environment as well as contributing to incomes and livelihoods (Morales and Wolff 2010; Ferraro and Andreatta 2014; Halloran and Vantomme 2014). However, more research and documentation are needed on their nutritional values in order to more efficiently promote insects as healthy food (Van Huis et al. 2013).

Research on edible insects in the United States and Western Europe is only starting to advance (Morales‐Ramos et al. 2013) with Europe focusing more on the use of insects as feed and less as food (Van Huis et al. 2013). In all regions of the world, long‐term preservation of edible insects has not been given much attention by both researchers and the food industry (Chidumayo and Gumbo 2010; Ferraro and Andreatta 2014) possibly because of their seasonal availability, and the small quantities harvested are consumed fresh. In sub‐Saharan Africa, many types of edible insects continue to be processed on a small scale by women and children for home consumption normally just before eating or sale in markets (Chidumayo and Gumbo 2010). However, there is growing interest in domesticating and making insects for food and feed readily available throughout the year.

*Ruspolia nitidula*, the edible grasshopper native to Uganda, has a nutritional and cherished cultural and economic importance (Van Huis et al. 2013; Martin 2014) to people of diverse cultures. In Uganda, *R. nitidula* is processed by either sautéing, deep frying, or boiling followed by drying. Processed *R. nitidula* are either consumed at home or commercially traded on a small scale mainly in the streets of Kampala city and other towns such as Masaka (Ageya et al. 2008). While small‐scale processing of grasshoppers in Uganda increases dietary diversity and nutrient intake and also contributes to incomes (Capinera 2008; Fellows 2009), it preserves the *R. nitidula* for only about 24 h.

This research, therefore, aimed at establishing the nutritional value of *R. nitidula* and developing preservation methods that extend its shelf life. The study specifically assessed the effect of harvesting season, geographical source area, and subtype on the nutritional composition of *R. nitidula*, and compared the effectiveness of two methods for preserving the sensory, microbiological, and chemical shelf stability of *R. nitidula*.

## Materials and Methods

### Sample preparation

Fresh *R. nitidula* was collected from Masaka and Kampala districts of Uganda in two subsequent swarming seasons of November–December and March–May. For each season, a 500‐g sample was washed using running portable tap water, thoroughly drained using a plastic colander, sorted based on subtype (color), and stored in clean and dry plastic containers at −18°C until further analyses.

### Nutritional composition analyses

Moisture content was determined by the draft oven method (Nielsen 2010), crude protein by the Kjeldahl (Horwitz 2001) method, fat content by the Soxhlet method (Nielsen 2010), total mineral content by ashing the *R. nitidula* in a carbolite furnace at 500 °C (Nielsen 2010), dietary fiber content by the acid detergent fiber assay, carbohydrate content by difference in nitrogen‐free extract (NFE) (Nielsen 2010), carotenoid content by the spectrophotometric method described in the HarvestPlus handbook of carotenoid analysis (Rodriguez‐Amaya and Kimura 2004), and potassium and phosphorus contents by wet digestion followed by spectrophotometry.

### Choice of the preferred cooking method

*Ruspolia nitidula* was prepared by sautéing, boiling, and deep frying. For each method, a different set of ingredients were added resulting in nine samples (Table 1), which were screened for sensory acceptability on a 9 point hedonic scale.

**Table 1:** Effect of preparation methods on the consumer acceptability of *Ruspolia nitidula*

Sample	Ingredients	Mean overall acceptability	Most frequent comments
Sautéed and dried			
A	Salt	5.667	Was dry
B	Salt, onion	**6.848**	The best, well spiced
C	Salt, onion, curry powder	6.121	Tastes good, dry, fatty
D	Salt, onion, curry powder, tomatoes	6.272	Well flavored
E	Salt, onion, curry powder, tomatoes, garlic	**6.576**	Sweet/good smell Bad garlic smell
Deep fried			
F	Salt, onion	5.272	Too oily
G	Salt	**5.515**	Very dry and no flavor
Boiled and dried			
H	Salt	6.709	So dry, but tasty
I	Salt, onion	7.000	Soft, tasty
J	Salt, onion, tomato	**7.226**	Best of all, well prepared

NB: 30‐panellist members were used.

### Preparation of boiled and sautéed *R*. *nitidula* for shelf‐stability studies

To 500 g of raw *R. nitidula*, 10 g salt and 40 g onion were added followed by boiling at 100°C for 30 min to a golden yellow color. A half of the boiled *R. nitidula* was sautéed without adding oil in a stainless steel pan over gentle heat for 30 min. The boiled and sautéed samples were separately dried at 80°C for 10 h in an air convection dryer (Innotech, D‐7115 Altdorf, Germany) to a moisture content of about 5%, allowed to cool to room temperature, and vacuum sealed. The vacuum packs for both types of samples were stored at room temperature in opaque paper bags to eliminate light. At 2‐week intervals, samples were monitored for stability over 12 weeks.

### Stability monitoring

#### Sensory stability of boiled and sautéed *R. nitidula*

Sensory acceptability of the sautéed and dried *R. nitidula* (A) and the boiled and dried *R. nitidula* (B) were evaluated by a panel of 30 regular consumers of *R. nitidula*. Each panelist was provided with five pieces of *R. nitidula* (A and B) to rate their acceptability of each attribute (aroma, color, taste, flavor, texture, appearance, and overall acceptability) on a 9‐point hedonic scale. This was done on a biweekly basis for 12 weeks.

#### Microbiological stability of boiled and sautéed *R. nitidula*

Total plate count (TPC) of boiled and sautéed *R. nitidula* samples was determined. A 30‐g sample was weighed aseptically, mixed with 90 mL of peptone water, and homogenized in a stomacher (Seward Stomacher, 400 circulator, England) to make a 10^−1^ dilution which was used to make subsequent dilutions. Each dilution was plated on sterile plate count agar (PCA) media in duplicate and incubated at 37 °C for 48 h.

### Fat rancidity monitoring based on acid value

Free fatty acids accumulation was determined (Nielsen 2010). A 5‐g sample was weighed into a clean conical flask and 50 mL 1:1 neutral mixture of ethanol–petroleum ether was added to dissolve the fat. The mixture was titrated with 0.07 mol/L ethanolic sodium hydroxide until the colorless phenolphthalein indicator turned pink. The titer value obtained was used to calculate the acid value expressed in mg KOH/g of the *R. nitidula* fat.

### Statistical analyses

IBM SPSS statistics for windows (Version 16, IBM Corporation, Armonk, NY) statistical software was used to compute the mean scores of the sensory quality and nutritional attributes. The 9‐point hedonic scale was used in the interpretation of the computed mean scores. ANOVA was used to determine the effect of season, sourcing geographical location, and subtype of nutritional composition at an alpha level of 0.05. Turkey's test was used for comparisons of the levels of the different factors where ANOVA indicated a significant difference. Excel (2007) was used to generate the graphs from results of microbial and chemical analyses.

## Results

### Effect of season on nutritional composition of *R. nitidula*

The nutritional composition of green and brown *R. nitidula* for the two harvesting seasons is presented in Table 2. Season had an effect (*P < *0.001) on moisture, dry matter, ash, fiber, and total carotenoids’ content of *R. nitidula*. Green and brown *R. nitidula* from the March–May season had a significantly lower moisture content (47–52%) and significantly higher dry matter content (48–52%) than brown and green *R. nitidula* from the November–December season, that is, 52–55% for moisture and 45–47% for dry matter. Green *R. nitidula* in the March–May season had the lowest moisture content and highest dry matter content, both of which were significantly different from the rest. *Ruspolia nitidula* from the March–May season had a significantly lower dietary fiber (11–12.2%) and significantly higher total carotenoids content (2084.8–2273.1 *μ*g/100 g) than *R. nitidula* from November to December season (13–14.5% for dietary fiber and 913.7–1389.4 *μ*g/100 g for total carotenoids). The ash content of green *R. nitidula* in the November–December season was the highest (3.97%) and significantly different from brown *R. nitidula* from the Kampala and green *R. nitidula* from the two geographical sourcing regions in the March–May season. Generally, protein (39–40.4%) content in the March–May season was slightly higher than that of the November–December season, 37.0–39.2%.

**Table 2:** Proximate, mineral, and total carotenoid contents of the green and brown *Ruspolia nitidula* subtypes for two swarming seasons

Subtype and Origin	Moisture	Nutritional components (% dry matter)							
		Crude protein	Crude fat	Carobhydrate	Dietary fiber	Ash	Phosphorus (mg/kg)	Potassium (mg/kg)	Carotenoids (*μ*g/100 g)
KGN	53.41 ± 0.9^b^	39.12 ± 1.5^a^	41.17 ± 0.7^a^	2.64 ± 0.8^a^	13.06 ± 0.8^a^	3.96 ± 0.4^b^	4.96 ± 0.3^a^	4.94 ± 0.4^a^	1267.1 ± 0.6^a^
KGA	47.63 ± 0.3^a^	40.08 ± 0.5^a^	42.37 ± 1.6^a^	3.02 ± 0.5^a^	11.89 ± 0.8^b^	2.67 ± 0.4^a^	4.96 ± 0.3^a^	5.08 ± 0.3^a^	2164.9 ± 3.1^b^
MGN	52.97 ± 1.0^b^	37.92 ± 1.4^a^	41.81 ± 2.2^a^	2.50 ± 0.4^a^	14.34 ± 0.5^a^	3.41 ± 0.7^a^	4.75 ± 0.2^a^	5.25 ± 0.1^a^	1389.4 ± 2.2^a^
MGA	47.56 ± 0.7^a^	40.30 ± 1.2^a^	42.23 ± 0.7^a^	3.21 ± 1.0^a^	11.32 ± 0.8^b^	2.60 ± 0.1^a^	4.75 ± 0.2^a^	4.80 ± 0.2^a^	2156.8 ± 2.5^b^
KBN	55.39 ± 2.4^b^	38.49 ± 0.7^a^	41.41 ± 1.0^a^	2.96 ± 1.6^a^	13.85 ± 1.3^a^	3.27 ± 0.5^a^	4.66 ± 0.4^a^	5.04 ± 0.3^a^	1224.5 ± 0.8^a^
KBA	51.60 ± 0.7^c^	39.73 ± 0.8^a^	42.38 ± 1.7^a^	2.96 ± 0.8^a^	12.19 ± 0.5^b^	2.74 ± 0.1^a^	4.85 ± 0.5^a^	5.55 ± 0.6^a^	2084.8 ± 2.8^b^
MBN	54.55 ± 2.7^b^	36.99 ± 0.9^a^	43.04 ± 1.5^a^	2.92 ± 1.6^a^	13.14 ± 0.9^a^	3.75 ± 0.1^a^	4.22 ± 0.4^a^	4.57 ± 0.5^a^	913.7 ± 0.1^c^
MBA	50.93 ± 0.9^c^	40.38 ± 2.6^a^	41.94 ± 2.0^a^	3.06 ± 0.4^a^	11.34 ± 0.7^b^	3.05 ± 0.5^a^	4.37 ± 0.3^a^	4.88 ± 0.6^a^	2273.1 ± 1.4^b^

Figures having the same superscripts in a column are not significantly different (*P* ≥ 0.05, three replicates).

M, Masaka; K, Kampala; G, green; B, brown; A, March–May season; N, November–December season.

### Effect of subtype on nutritional composition of *R. nitidula*

*Ruspolia nitidula* subtype did not have an effect on macro‐ and micronutrient compositions except moisture and dry matter. Brown *R. nitidula* had a significantly lower dry matter (48–49%, *P* = 0.018) and higher moisture (51–52%, *P* = 0.021) content compared to green *R. nitidula* (~48% for moisture and ~52% for dry matter).

### Effect of sourcing geographical location on nutritional composition of *R. nitidula*

*Ruspolia nitidula* sourcing geographical location did not have any effect on nutrient composition except for phosphorus content (*P = *0.001). Brown *R. nitidula* from the Kampala harvested in the November season had a significantly higher phosphorus content (5.55 mg/kg) than brown *R. nitidula* from the Masaka in the April season (4.56 mg/kg). However, phosphorus content of the above was not different from that of other brown and green subtypes in the two seasons.

### Sensory preference of *R. nitidula* processed by three methods

Acceptability can be an indirect measure of preference, implying that the sample with a higher acceptability is preferred. Generally, for both sautéed and boiled samples, the mean overall acceptability increased as the number of spices added increased (Table 1). In general, boiled and dried samples were most preferred and had the highest mean overall acceptability scores (6.7–7.2), followed by sautéed and dried samples (6.1–6.85), and finally deep fried samples (5.2–5.5). The boiled and dried samples with salt, onions, and tomatoes had the highest mean overall acceptability score of 7.2, followed by another boiled and dried sample with salt and onions only, with a similar overall acceptability score of 7. The two were followed by the sautéed and dried subsample with salt and onions only, with an overall acceptability score of 6.85. On the 9‐point hedonic scale, these three samples with the highest mean overall acceptability were liked moderately.

### Shelf life of processed *R. nitidula*

#### Sensory stability

Sensory acceptability scores of sautéed and boiled dried samples during the 12 weeks are presented in Tables 3 and 4. Sensory acceptability of sautéed and dried *R. nitidula* did not change significantly over the 12 weeks (Table 3). On the other hand, sensory acceptability of the boiled and dried *R. nitidula* dramatically and significantly decreased by the second week (Table 4) to about 5 (indifferent) which was maintained until week 12.

**Table 3:** Changes in mean attribute scores of sautéed and dried *Ruspolia nitidula* flavored with salt and onions over 12 weeks of storage

Attribute	Biweekly mean attribute score						
	0	2	4	6	8	10	12
Aroma	6.9 ± 2.34^a^	6.8 ± 1.21^a^	6.9 ± 1.25^a^	7.3 ± 1.17^a^	6.6 ± 1.52^a^	7.3 ± 0.99^a^	6.6 ± 1.52^a^
Taste	7.6 ± 1.4^a^	7.1 ± 1.36^a^	7.2 ± 1.37^a^	7.6 ± 1.13^a^	7.3 ± 1.02^a^	7.9 ± 0.86^a^	7.3 ± 1.02^a^
Flavor	7.7 ± 1.92^a^	7.0 ± 1.18^a^	7.0 ± 1.18^a^	7.4 ± 1.28^a^	6.9 ± 1.14^a^	7.0 ± 1.44^a^	6.9 ± 1.14^a^
Texture	7.0 ± 2.13^a^	6.5 ± 1.72^a^	6.5 ± 1.50^a^	6.7 ± 1.81^a^	6.6 ± 1.61^a^	7.0 ± 1.21^a^	6.6 ± 1.61^a^
Appearance	6.8 ± 2.41^a^	6.2 ± 1.89^a^	6.6 ± 1.74^a^	6.8 ± 1.72^a^	6.9 ± 1.35^a^	7.3 ± 1.84^a^	6.9 ± 1.34^a^
Color	7.1 ± 1.16^a^	7.4 ± 1.22^a^	7.0 ± 1.07^a^	7.1 ± 0.77^a^	6.9 ± 1.31^a^	7.7 ± 1.23^a^	6.9 ± 1.30^a^
Overall acceptability	7.2 ± 1.18^a^	6.8 ± 1.33^a^	6.9 ± 1.46^a^	7.2 ± 1.39^a^	6.9 ± 1.28^a^	7.3 ± 1.18^a^	6.9 ± 1.40^a^

Results are mean ± standard deviations of 30 panelists per sitting for a period of 12 weeks.

Figures having the same superscripts are not significantly different (*P* ≥ 0.05, three replicates).

**Table 4:** Changes in mean attribute scores of boiled and dried *Ruspolia nitidula* flavored with salt and onions over 12 weeks of storage

Attribute	Biweekly mean attribute score						
	0	2	4	6	8	10	12
Aroma	5.8 ± 1.32^a^	5.0 ± 2.52^b^	5.1 ± 1.98^b^	5.0 ± 1.47^b^	5.3 ± 1.56^b^	5.7 ± 1.44^a^	5.3 ± 1.78^b^
Taste	6.6 ± 1.68^a^	5.2 ± 1.92^b^	6.0 ± 1.82^b^	5.2 ± 1.61^b^	5.0 ± 2.12^b^	5.7 ± 1.55^b^	5.5 ± 1.81^b^
Flavor	6.5 ± 1.51^a^	5.4 ± 1.69^b^	5.1 ± 1.79^b^	5.0 ± 1.83^b^	5.7 ± 1.69^b^	5.6 ± 1.30^b^	5.0 ± 2.22^b^
Texture	6.2 ± 1.43^a^	5.5 ± 2.33^b^	5.9 ± 2.04^b^	5.7 ± 1.58^b^	5.7 ± 1.78^b^	5.9 ± 2.08^a^	5.2 ± 1.95^b^
Appearance	6.8 ± 1.52^a^	4.7 ± 1.95^b^	6.0 ± 1.85^b^	5.2 ± 1.79^b^	5.1 ± 1.99^b^	5.5 ± 1.87^b^	5.1 ± 2.10^b^
Color	6.4 ± 1.99^a^	5.4 ± 1.96^b^	5.4 ± 1.92^b^	5.7 ± 1.76^b^	5.3 ± 1.89^b^	5.8 ± 1.69^b^	5.2 ± 1.93^b^
Overall acceptability	6.4 ± 1.59^a^	5.2 ± 1.71^b^	5.6 ± 1.62^b^	5.3 ± 1.58^b^	5.4 ± 1.86^b^	5.7 ± 1.43^b^	5.1 ± 1.91^b^

Results are mean ± standard deviations of 30 panelists per sitting for a period of 12 weeks.

Figures having the same superscripts are not significantly different (*P* ≥ 0.05, three replicates).

#### Microbiological stability

**Figure 1:**
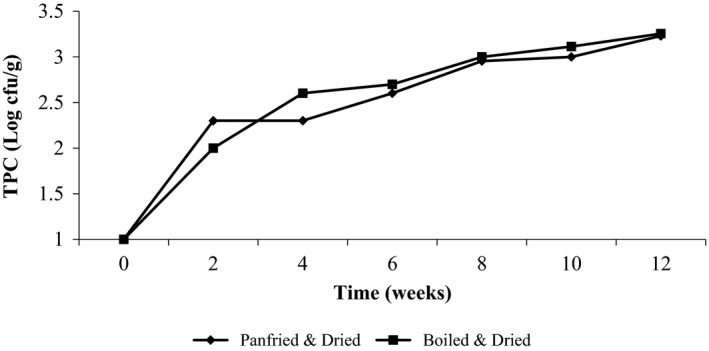
Changes in the total plate count of *Ruspolia nitidula* during 12 weeks of storage at ambient temperature.

The microbiological quality of *R. nitidula* with time was monitored by the TPC (Fig. 1). From week 4 onward, boiled and dried samples had a slightly higher total plate count than sautéed and dried *R. nitidula*. The highest count observed was 3.2 log cfu/g.

#### Chemical stability (acid value)

**Figure 2:**
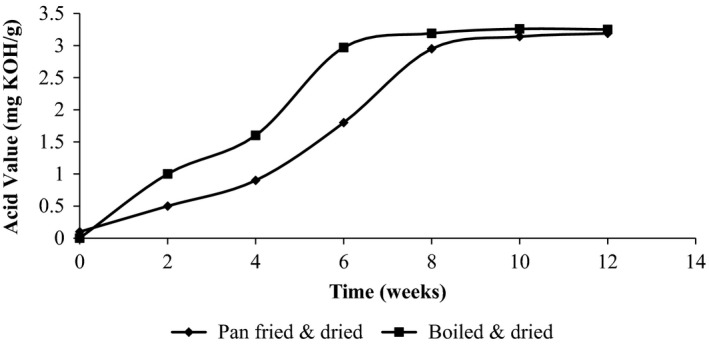
Changes in the acid value of *Ruspolia nitidula* during 12 weeks of storage at ambient temperature.

The acid values of both boiled and dried and sautéed and dried *R. nitidula* increased with time and stabilized at about 3.3 mg KOH/kg (Fig. 2). For boiled and dried *R. nitidula*, the acid value stabilized by 6th week, while for sautéed and dried *R. nitidula* it stabilized by the 8th week.

## Discussion

### Effect of harvesting season on the nutritional composition of *R. nitidula*

Season had an effect on moisture, dry matter, dietary fiber, and total carotenoid content of *R. nitidula*. Being herbivores (Hahn and Orrock 2015; Lenhart et al. 2015), *R. nitidula* feed on crops and grass, implying that their nutritional composition is affected by their feed among other factors. *Ruspolia nitidula* normally swarms in the rainy seasons of March–May and November–December. It is therefore possible that the effect of season on moisture, dry matter, dietary fiber, and total carotenoid was due to differences in the type of feed sources available and fed on by the *R. nitidula* in these seasons. Due to the absence of conclusive research on factors affecting feed intake and nutritional composition of *R. nitidula*, the effect of feed and other factors affecting nutritional composition are not clearly known.

### Effect of sourcing geographical location and subtype on the nutritional composition of *R. nitidula*

Subtype had a significant effect on moisture and dry matter content, while sourcing geographical location had a significant effect on mineral content, particularly phosphorus. The observed differences were inconsistent with regard to the macronutrients and minerals. It is therefore possible that these differences in phosphorus, moisture, and dry matter content are purely due to individual differences and not necessarily the influence of the subtype or sourcing geographical location. The fact that the differences in the moisture and dry matter content did not induce a change in the macronutrient composition (protein, fat, carbohydrate, and dietary fiber) substantiates the possibility that the observed differences are random and individual. There is therefore no conclusive evidence that subtype and sourcing geographical location have an influence on the nutritional composition of *R. nitidula*.

### Nutrient composition of *R. nitidula*

*Ruspolia nitidula* are very nutritious with 36–40% protein, 41–43% fat, 10–13% dietary fiber, 900–2300 *μ*g/100 g carotenoids, 3–4% ash, and 4–6 mg/kg potassium and phosphorus (Table 2). The moisture content (47–55%) is less than other major protein sources such as fish with 80% and meat muscle with 62–70%. Thus, *R. nitidula* has a much higher dry matter content (45–53). This implies that on a dry matter basis, *R. nitidula* does not only contain more edible nutrient percent, it is also a more concentrated source of nutrient than meat and fish.

The protein content (36–40.4%) recorded for *R. nitidula* is higher than that of the small grasshopper (14.6%) and the large grasshopper (20.6%) species; quite close to *Ruspolia differens* (43–44%) in Kenya; comparable to winged termites (33.51–39.74%) in western Kenya and other edible insects. Notably, the protein content of *R. nitidula* is higher than that of commonly consumed animal and plant protein sources such as veal, lamb, chicken, and herring fish (7.5–23%); milk and milk products (3–26%); whole eggs (13%); common beans (~24%); peas (~23%); and comparable to that of soy beans ~38%. Insects are therefore high‐potential sources of protein to the population. While meat, milk, and egg protein are of higher biological value and digestibility compared to plant protein, the biological value and digestibility of *R. nitidula* have not been determined and therefore, need to be established for an informed comparison.

The fat content (41–43%) is also higher than that of other grasshopper species such as small (6.1%) and large grasshoppers (3.3%), as well as the 14 edible insects in western Nigeria including a grasshopper species, *Zonocerus variegatus* (3.8%), but comparable to the winged termites (44–47%) in Kenya. *Ruspolia nitidula* also has a markedly higher fat content than that of meat, pork, and fish, all of which average less than 22% fat. Mbabazi et al. (2009) reported that the lipid/fat from *R. nitidula* consist of over 58% unsaturated fatty acids with oleic acid (41%) and linolenic acid being the predominant mono‐ and polyunsaturated fatty acids, respectively. Although no further studies have confirmed these findings, the high content of unsaturated fatty acids indicates that *R. nitidula* not only contains predominantly healthy fat, but it is also highly likely to suffer spoilage from oxidative rancidity.

The carbohydrate content (2.5–3.06%) is comparable to the small grasshoppers (3.9%), the large grasshopper (2.2%), and the winged termites (0.72–8.73%) in Kenya, but lower than that of *Z. variegatus* (63.2%), a grasshopper species in south‐western Nigeria. Like animals and fish, *R. nitidula*'s carbohydrate content is much lower than that of plant food sources such as pulses and legumes that average between 24–68% and 68–80% for cereals.

*Ruspolia nitidula* contains 11–13% dietary fiber, higher than the 14 edible insects (1.68–3.40%) in south‐western Nigeria and 4 winged termites (6.37–7.21%) in Kenya. Dietary fiber is normally found in only plant foods such as fruits, vegetables, nuts, and grains and not in meat, milk, or eggs. The dietary fiber of *R. nitidula* is higher than that of common plant sources such as peas (4.7%), sesame seeds (7.9%), and fruits such as mango (2.4%), guava (3.7%), and avocado (3.4%). Dietary fiber is important for normal bowel function and may play a role in the prevention of chronic diseases such as cancer, coronary artery disease, and diabetes mellitus. Being both a good source of dietary fiber and protein is a unique and excellent nutritional property compared to plant and animal nutrient sources.

Two minerals were analyzed, potassium and phosphorus with amounts ranging from 4 to 6 mg/kg. These values are much less than those of major plant and animal sources. The total carotenoid content ranged from 900 to 2300 *μ*g/100 g or 75 to 192 RE/100 g based on retinol equivalents (RE) of 12 *μ*g for *α*‐carotene, *β*‐cryptoxanthin, and other provitamin A carotenoids. This is generally much higher than that of other edible grasshopper species such as *Z.  variagatus* (6.8 *μ*g/100 g). Compared to other animal sources, muscle meats hardly contain any vitamin A except in the liver (~13,877 *μ*g/100 g) to which *R. nitidula* can only be compared. This implies that *R. nitidula* is a potential source of carotenoids and thus vitamin A.

### Screening for the best preparation method of *R. nitidula*

**Figure 3:**
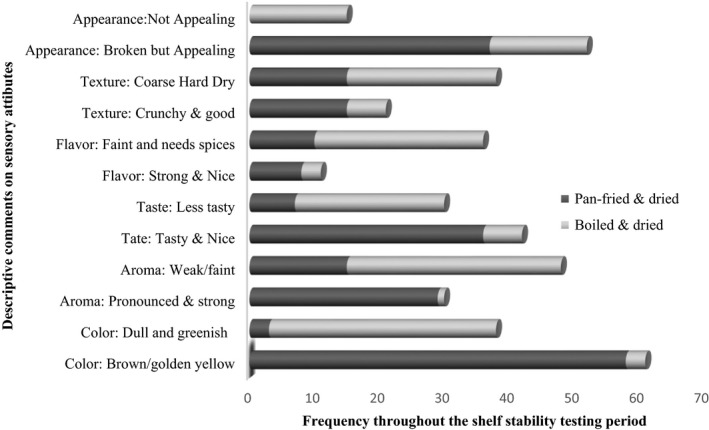
Frequency of descriptive comments given by panelists on each attribute during shelf‐life testing of *Ruspolia nitidula*.

The hedonic scale is normally used for expression of opinion/preference through acceptance testing. From Table 1, the mean overall acceptability on a 9‐point hedonic scale generally increased on addition of spices from only salt to onions, tomatoes, curry powder, and garlic. However, there was a notable exception in the sautéed samples, where addition of onions to *R. nitidula* already containing salt increased the acceptability from 5.7 to 6.8. Further addition of curry powder and tomatoes did not lead to any further increment in the mean overall acceptability, but rather a decrease from 6.8 to 6.1 and 6.3. Addition of garlic seemed to reverse the trend (6.6), but could not elicit the same effect as salt and onion alone. This suggests that adding curry powder and tomatoes probably did not suit the expected sensory characteristics and therefore scored less than the samples to which they were not added. For both boiled and sautéed samples, subsamples with salt and onions had almost the same score (I = 7 and B = 6.8), and close to the highest score. Unlike samples with salt and onions only, further addition of spices elicited mixed responses that could possibly be due to a mere expression of liking or disliking of the spices rather than the products. Therefore, sample B (sautéed and dried) and I (boiled and oven dried) were chosen for shelf‐life testing. This is because they were liked by both consumers who enjoy spices and those who do not. According to comments from panelists (Fig. 3), being moderately dry, quite soft, and not oily were some of the characteristics associated with the products’ preference.

### Shelf life of processed *R. nitidula* products

#### Sensory acceptability and shelf stability of *R. nitidula*

Loss of consumer acceptance is a common criteria used to monitor shelf life of foods. Often, the shelf life of products stored at ambient temperature is based on sensory quality (Kilcast 2010). During the 3 months of evaluation, both sautéed (A) and boiled (B) *R. nitidula* with salt and onion were all liked moderately, and none was disliked on average. None of the sensory attributes was on average liked extremely for both preservation methods.

Generally, the sautéed and dried *R. nitidula* scored higher (6.5–7.6) for each attribute on average and had the highest mean overall acceptability score (6.9–7.2) compared to boiled and dried *R. nitidula* (5.1–6.4). The sautéed and dried *R. nitidula* (A) remained acceptable throughout the testing period with a mean overall acceptability score ranging between 6.8 and 7.2, that is, generally liked moderately. Texture was the least liked attribute scoring averagely, 6–7. Majority of the sensory panelists commented that sautéed and dried *R. nitidula* had a golden yellow appearance, marked/strong flavor, was tasty, coarse and crunchy, and dry/not oily (Fig. 3). Although some liked the crunchiness, they did not like the other texture attributes such as being coarse and dry. These could be the reasons as to why sautéed and dried *R. nitidula* never attained a score of 8 (like very much) or 9 (like extremely). Appearance could have scored less because the sautéed and dried *R. nitidula* were broken. This was due to pressure applied during vacuum packaging of the dried samples. The breakage was worsened by double packing, that is, use of double polyethylene packs, one inserted into another. The double packing was necessary because a single pack was often pierced by the *R. nitidula* mandibles leading to loss of vacuum.

Boiled and dried *R. nitidula* had the least mean overall acceptability scores ranging between the “indifferent” (5.0) to “almost like moderately” (6.6). Notably, all the attributes were initially scored about 7 (like moderately), quite close to the sautéed and dried scores, but by the second week, the scores declined and stabilized at about 5. This trend can be partly explained by panelists’ comments about the different sensory attributes. The color of the boiled and dried *R. nitidula* remained greenish and dull. This was disliked by many panelists compared to the brown (golden yellow) color of sautéed and dried *R. nitidula*. This could have influenced the scoring of other attributes as well, because color is known to affect the consumers’ perception of all the other sensory attributes (Lawless and Heymann 2010). Color is a key factor in consumers’ perception, choice, and preference for food as it predetermines the expected perception of flavor and taste (Socaciu 2007; Lamb et al. 2008). This is probably contributed to the majority of the panelists commenting that boiled and dried R*. nitidula* had a faint aroma and flavor, was less tasty, and not appealing.

In general, the sautéed and dried *R. nitidula* was preferred to the boiled and dried *R. nitidula* because of the differences in color, aroma, taste, and appearance. The difference in preference of the sautéed and dried *R. nitidula* compared to boiled and dried *R. nitidula* can be attributed to the method of preparation and its effect on final product sensory quality. Frying operations normally produce food with an attractive color, well‐developed flavor, and good aroma (Keeling and Ridout 2002; Du Toit and Botha 2007), consistent with panelists’ comments in Figure 3. Sautéing is most likely to have led to the more pronounced/strong good flavor and aroma, the golden brown color, and good taste.

None of the samples had attribute scores below 5, however, sautéed and dried *R. nitidula* was preferred to boiled and dried *R. nitidula*. Sautéed and dried *R*. *nitidula* can therefore be improved to eliminate the undesirable coarseness, hardness, and dryness (Fig. 3), possibly by optimizing the drying temperature–time combination. In light of this, there is need to establish a sensory quality specification such as a cut‐off point or a defined point on a hedonic scale to cater for product quality and the effects of processing changes on shelf life (Hough 2010).

### Microbial stability of *R. nitidula*

Ready‐to‐eat dried *R. nitidula* remained microbiologically stable during the 12 weeks. The Canadian guidelines for microbiological quality of ready‐to‐eat foods that need no further preparation prior to consumption recommends a TPC of less than 10^5^ log cfu/g for a food to remain acceptable. A similar guideline by the New South Wales state's (2009) permits the same value but for fully cooked foods for immediate sale or consumption. In view of the above criteria, the highest count observed was 3.2 log cfu/g (Fig. 1), a value less than 5 log cfu/g, and hence regarded safe for human consumption. Although the United Kingdom and Hong Kong microbiological criteria recommend the same threshold value (5 log cfu/g), but disregard TPC as an applicable criteria for judging the quality of dried foods (HPA 2009, Centre for Food Safety, Food and Environmental Hygiene Department 2014). The available guidelines are based on meat and fish products, whose nutritional composition (intrinsic environment) differs from that of *R. nitidula* (exemplified by the high protein [36–40%], fat [41–43%], and low carbohydrate [2–3%] contents) implying that also the microbial ecology is different (Ray and Bhunia 2013). There is need therefore to study *R. nitidula*'s microbial ecology and set microbiological limits regarding the different types of microorganisms in ready‐to‐eat *R. nitidula* that are of concern in guaranteeing its quality and safety as food (King 2013).

### Chemical stability of *R. nitidula*

Hydrolytic rancidity results into formation of free fatty acids which can contribute to objectionable doors and flavors (O'Brien 2008). The acid value increased for the first 3 weeks, stabilizing by the 6th week at about 3.2 mg KOH/g of sample. Generally, high acid values above 2 mg KOH/g are correlated with rancidity, since the free fatty acids may have off odors or may undergo auto oxidation to produce off flavors and odors (Fennema 1996; Nielsen 2010).

However, though the amount of free fatty acids was high, rancid flavors or aromas were not detected or reported by the sensory panelists. *Ruspolia nitidula* fat has 41% linolenic acid, 31% palmitic acid, and 13% linolenic acid all of which have carbon atom number greater than 12–14 carbon atoms known to contribute to off flavors upon hydrolysis. Even though the contribution of free fatty acids to autoxidation is insufficient and contradictory, inability of panelists to detect rancid flavors and aromas may not necessarily imply its absence. This is because there was no direct measurement of primary and secondary auto‐oxidative products responsible for off flavors and aromas. It is possible that by this time, they existed in lower concentrations than the sensory thresholds detectable by panelists. This possibly explains why FFA is only used to accompany other auto‐oxidative and stability measurements. Therefore, in any further attempts to determine shelf life of the highly fatty *R. nitidula*, measurements of primary and secondary oxidation products should be considered.

## Conclusion

*Ruspolia nitidula* is highly nutritious compared to conventional foods and has potential to significantly contribute to food and nutrition security. Proper processing methods can guarantee *R. nitidula* consumers a safe and a tasty product beyond the harvesting season.

### Recommendation

There is need to fully profile and characterize the nutrients in *R. nitidula* so as to understand their functionality and bioactivity, as well as characterize its microbial ecology. Alternatives to vacuum packaging should be investigated to better preserve the structure of processed *R. nitidula* products and extend the shelf life beyond the 3 months attained in this work. Primary and secondary oxidation products measurements should be done to directly account for oxidative rancidity and its contribution to sensory quality and stability.

## Conflict of Interest

None declared.
